# High-performance macromolecular data delivery and visualization for the web

**DOI:** 10.1107/S2059798320014515

**Published:** 2020-11-26

**Authors:** David Sehnal, Radka Svobodová, Karel Berka, Alexander S. Rose, Stephen K. Burley, Sameer Velankar, Jaroslav Koča

**Affiliations:** a CEITEC – Central European Institute of Technology, Masaryk University, Kamenice 753/5, 625 00 Brno, Czech Republic; b National Centre for Biomolecular Research, Faculty of Science, Masaryk University, Kamenice 753/5, 625 00 Brno, Czech Republic; c Protein Data Bank in Europe (PDBe), European Molecular Biology Laboratory, European Bioinformatics Institute (EMBL–EBI), Wellcome Genome Campus, Hinxton CB10 1SD, United Kingdom; d Regional Centre of Advanced Technologies and Materials, Department of Physical Chemistry, Faculty of Science, Palacký University Olomouc, Šlechtitelů 241/27, 779 00 Olomouc, Czech Republic; e Research Collaboratory for Structural Bioinformatics (RCSB), San Diego Supercomputer Center, University of California San Diego, 9500 Gilman Drive, La Jolla, San Diego, CA 92093-0743, USA; f RCSB Protein Data Bank, Institute for Quantitative Biomedicine and Department of Chemistry and Chemical Biology, Rutgers, The State University of New Jersey, 174 Frelinghuysen Road, Piscataway, NJ 08854-8076, USA; g Cancer Institute of New Jersey, Rutgers, The State University of New Jersey, 195 Little Albany Street, New Brunswick, NJ 08903-2681, USA; h RCSB Protein Data Bank, San Diego Supercomputer Center and Skaggs School of Pharmacy and Pharmaceutical Sciences, University of California San Diego, 9500 Gilman Drive, La Jolla, CA 92093-0654, USA

**Keywords:** data delivery, visualization, macromolecules, browser-based, web-based

## Abstract

Biomacromolecular structural data make up a vital and crucial scientific resource that has grown not only in terms of its amount but also in its size and complexity. Furthermore, these data are accompanied by large and increasing amounts of experimental data. Additionally, the macromolecular data are enriched with value-added annotations describing their biological, physicochemical and structural properties. Today, the scientific community requires fast and fully interactive web visualization to exploit this complex structural information. This article provides a survey of the available cutting-edge web services that address this challenge. Specifically, it focuses on data-delivery problems, discusses the visualization of a single structure, including experimental data and annotations, and concludes with a focus on the results of molecular-dynamics simulations and the visualization of structural ensembles.

## Abstract 2

This article provides a survey of available web services and tools for data delivery and visualization of macromolecular structures.

## 1. Introduction   

Biomacromolecular structural data, originating from more than seven decades of intensive research, form a highly valuable and scientifically vital resource that provides a mechanistic understanding of biological systems. Currently, more than 166 000 experimentally determined three-dimensional structures of biological macromolecules are available from the open-access Protein Data Bank (PDB) that is jointly managed by the Worldwide Protein Data Bank consortium (wwPDB Consortium, 2019 ▸), with 200–300 new structures being added every week. Biomacromolecular structural data (atomic coordinates) obtained from macromolecular crystallography (MX) or electron microscopy (3DEM) are accompanied by experimental data: measured structure factors yielding electron-density maps or directly measured Coulomb electric potential maps. NMR entries in the PDB are represented by ensembles of 10–20 structures, reflecting the uncertainty in the structural model estimated from experimentally derived restraints.

**Figure 1:**
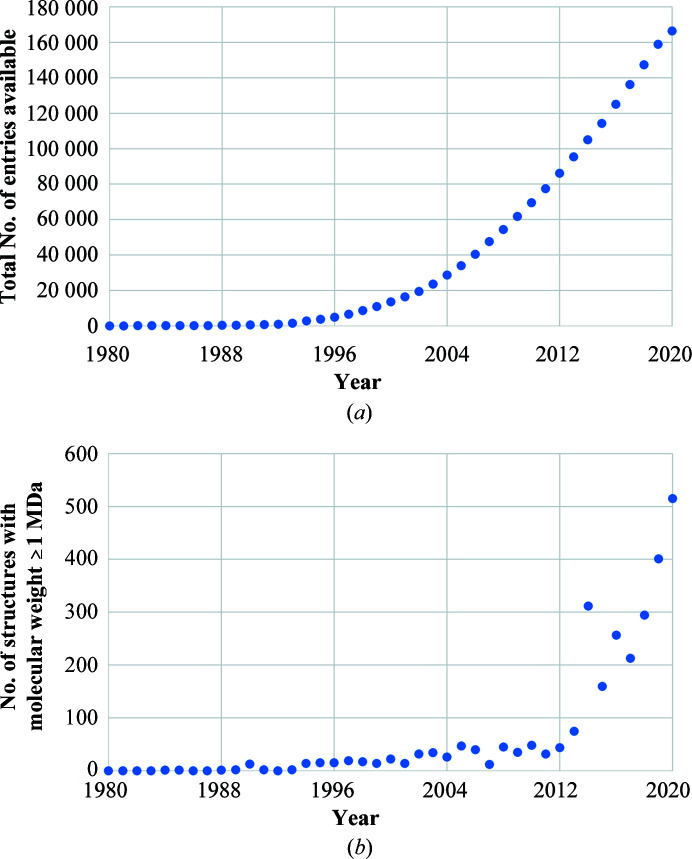
(*a*) Growth in the size of the PDB Core Archive. (*b*) The growing number of structures in the PDB Core Archive, grouped by their year of release, with a molecular weight of their preferred assembly that is greater than or equal to 1 MDa.

**Figure 2:**
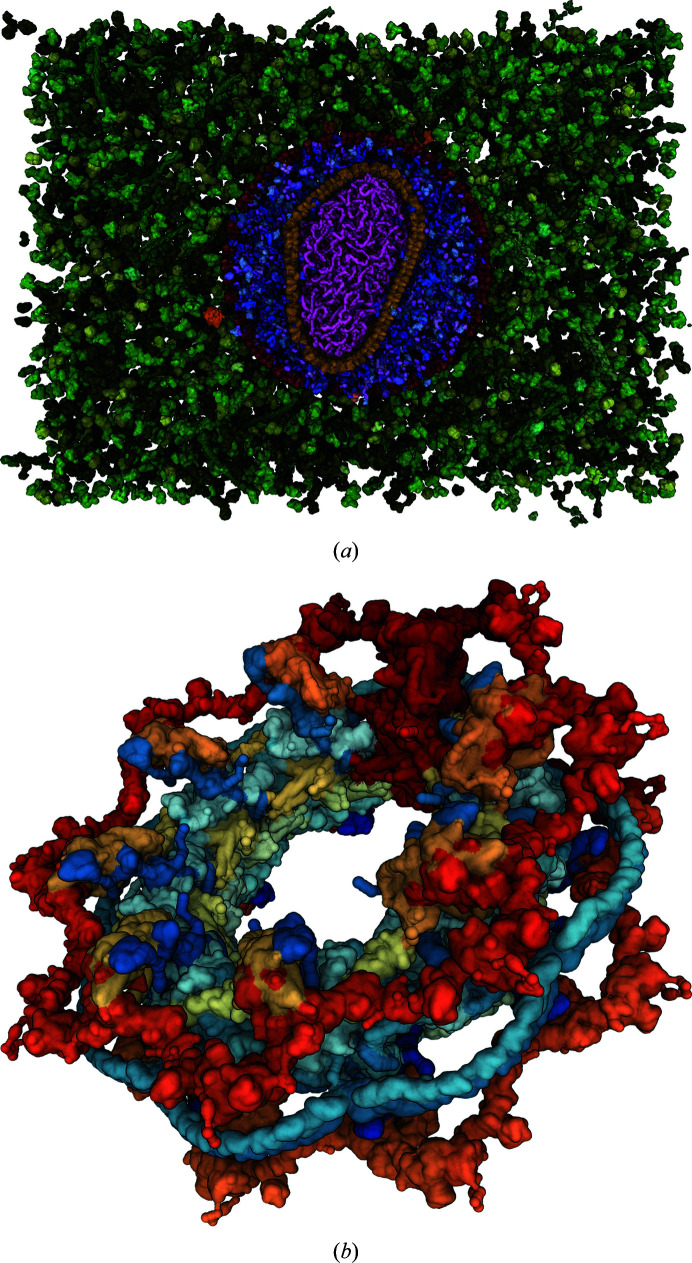
(*a*) *CellPACK* model of enveloped HIV capsid in blood serum (68 million atoms) visualized in a web browser using Mol*. (*b*) The nuclear pore complex (PDB-Dev ID PDBDEV_00000012) with 243 000 residues (represented as coarse elements) visualized in a web browser using Mol*.

The PDB archive continues to grow both in terms of the number of new entries (count of structures; see Fig. 1 ▸ *a*) and also in the complexity and size of the structures deposited in the PDB (the size of individual structures; see Fig. 1 ▸ *b*). The largest single entry is the HIV-1 capsid (PDB entry 3j3q), with 2.4 million atoms (Zhao *et al.*, 2013 ▸). However, the entries in the PDB also serve as building blocks for much larger models of biological systems, such as the *cellPACK* (Johnson *et al.*, 2013*a* ▸,*b* ▸, 2015 ▸) model of the HIV-1 capsid in blood serum (Johnson *et al.*, 2014 ▸), with nearly 68 million atoms, as shown in Fig. 2 ▸(*a*). The PDB-Dev (Johnson *et al.*, 2013*a* ▸,*b* ▸, 2014 ▸) prototype system collects gigantic structural models obtained using integrative/hybrid modelling; for example, the nuclear pore complex (PDB-Dev ID PDBDEV_00000012; Kim *et al.*, 2018 ▸), which contains 243 000 residues (represented as coarse elements; see Fig. 2 ▸ *b*).

Value-added annotations provide the necessary biological context for the macromolecular structure data. The increasing amount of value-added annotations include information about many biological properties (mutation positions and effects, functional and active sites, channels and pores *etc.*), physicochemical properties (charges, flexibility *etc.*) and structural properties (ligand binding, various quality criteria *etc.*). These data are stored in multiple databases, and many of them are collected in the PDBe-KB database (PDBe-KB Consortium, 2020 ▸) and the RCSB PDB database (Goodsell *et al.*, 2020 ▸). Moreover, not surprisingly, the number of available annotation types is also growing.

Biological macromolecules are inherently dynamic in nature, and hence the research community is not only interested in the static data available in the PDB but also generates information on the dynamics of the structures using molecular dynamics and, increasingly, experimental techniques such as electron microscopy, X-ray free-electron lasers and serial crystallography. These data are the basis of various complex analyses and examinations providing mechanistic insights into the function of biological macromolecules.

Visualization of these scientific data is pivotal in the analysis of biomacromolecular structures by the broader scientific user base, with most non-structural biology users expecting access to these data via web-based visualization tools. Specifically, researchers require a fast, responsive and fully interactive web visualization of all of the data mentioned above: atomic coordinates, experimental data, annotations and molecular dynamics.

Here, we describe the currently available cutting-edge web services that address these challenges.

Firstly, we focus on data-delivery problems. We then discuss the visualization of a single structure, including experimental data and annotations, and conclude with a focus on dynamic data and the visualization of structural ensembles.

For alternative surveys of tools for molecular visualization, we direct the reader to Martinez *et al.* (2020 ▸) and Miao *et al.* (2019 ▸).

## 2. Methods   

### 2.1. Data delivery: coordinates   

Until now, the naïve approach of delivering the complete coordinate model worked owing to the modest size of the macromolecular structures studied using traditional structure-determination methods. This approach was used successfully even when only a small part was visualized, such as a binding site or a cartoon representation of a backbone without side chains. With rapid advances in structure-determination techniques, increasingly large macromolecular machines are within the reach of structure-determination studies (Zhao *et al.*, 2013 ▸; Kim *et al.*, 2018 ▸). The naïve approach of delivering the complete coordinate model is increasingly inadequate because of its low performance for large systems. To alleviate this issue, advanced approaches such as selective data delivery are required.

One approach is to precompute static subsets of data on the server for delivery, but this would require every possible combination to be precomputed, making this approach unfeasible. Another more practical approach is to create a system with a dynamic query language to compute the subsets of macromolecular structures on the fly (Sehnal, Pravda *et al.*, 2015 ▸) for efficient data delivery. This second approach was further developed in the *LiteMol* suite (Sehnal *et al.*, 2017 ▸), which includes a module called *CoordinateServer*. *CoordinateServer* performs an on-the-fly selection of several critical parts of the biomacromolecule; for example, backbone, side chains, heteroatoms, atoms necessary for cartoon model visualization, chains, residues and ligands including their surroundings *etc.* Based on the user request, a relevant subset of atoms is selected by *CoordinateServer* and delivered for further analysis or visualization. The same approach of selective delivery is also implemented in Mol* (Sehnal *et al.*, 2018 ▸). *CoordinateServer* (and its Mol* successor *ModelServer* 1) is available for use by any software that supports the standard mmCIF format for the storage and efficient delivery of macromolecular structure data.

**Figure 3:**
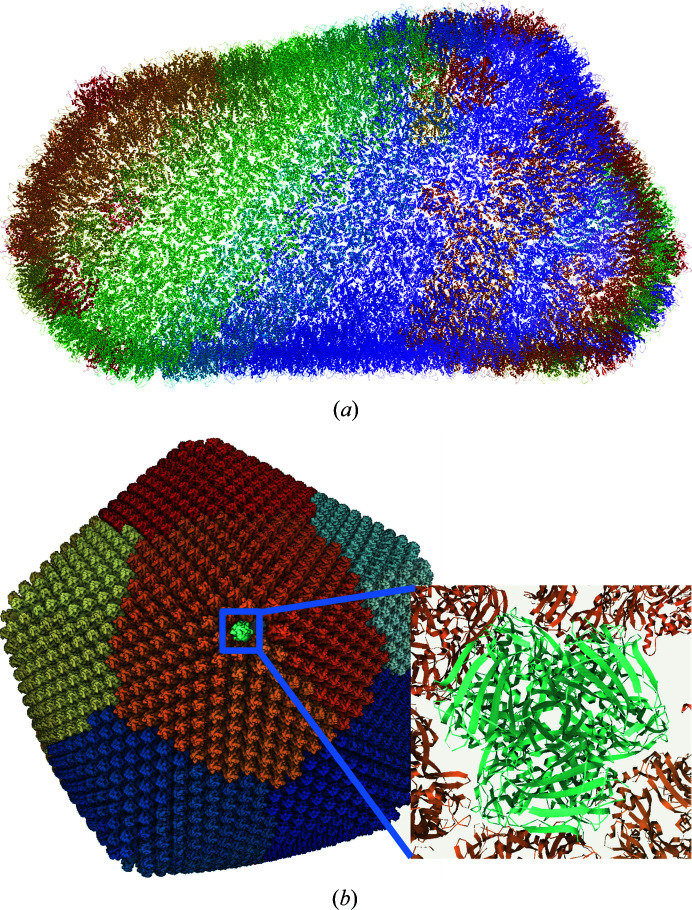
(*a*) *LiteMol* suite visualization of the HIV-1 capsid (PDB entry 3j3q). The HIV-1 capsid contains 2.44 million atoms, and its gzip-compressed mmCIF file is 41.78 MB in size. To display a structure utilizing a cartoon representation, only a subset of backbone atoms is required, reducing the size to 1.54 MB in BinaryCIF. (*b*) Mol* visualization of faustovirus (PDB entry 5j7v; Klose *et al.*, 2016 ▸). The faustovirus assembly has 40 million atoms. A naïve approach requires ∼480 MB of memory to represent the *XYZ* positions as 32-bit floats. The advanced data structures in Mol* allocate only 50 MB of memory to represent and visualize the whole structure by utilizing the 2760-fold symmetry of the structure.

Further improvements were made to the data-delivery process by introducing novel coordinate-file formats requiring markedly less space than standard PDBx/mmCIF and PDB files; namely, the MMTF format (Bradley *et al.*, 2017 ▸) and the BinaryCIF format (originally introduced as part of the *LiteMol* suite), which was later integrated into Mol*. Fig. 3 ▸(*a*) shows a marked reduction in the size of the data delivered for the visualization of the HIV-1 capsid via the *LiteMol* suite.

In parallel, enhancements can be achieved by utilizing an efficient in-memory representation of the macromolecular structure. Fig. 3 ▸(*b*) illustrates the efficient memory usage in Mol* that is necessary to represent large viral assemblies.

### 2.2. Data delivery: volumetric data (electron-density and electric potential maps)   

**Figure 4:**
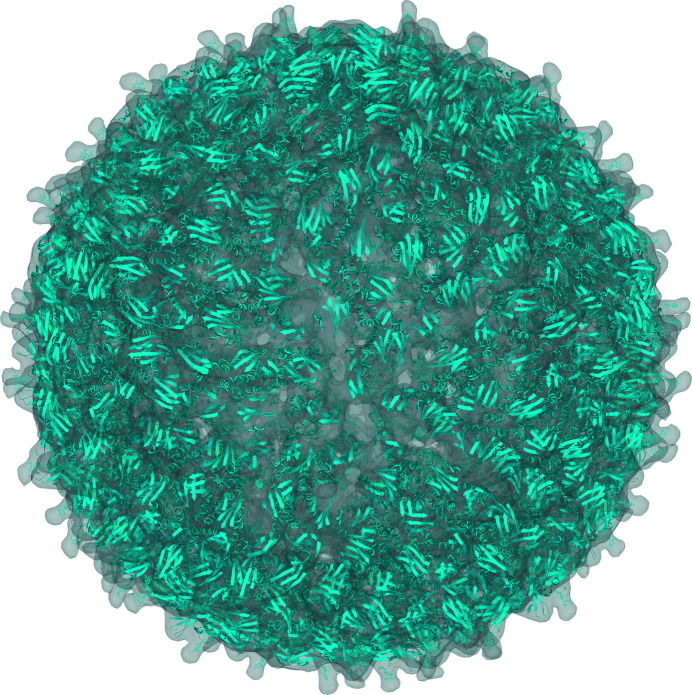
Reduction of data delivery for Zika virus (PDB entry 5ire) in the *LiteMol* suite by the application of *DensityServer*. The data size is reduced from 1.6 GB to 1 MB by lowering the resolution while maintaining visual fidelity. For a live version, see https://v.litemol.org/?example=zika-cryo-em.

Volumetric data such as electron-density or electric potential maps are markedly larger than coordinate files. For example, the Zika virus capsid (PDB entry 5ire) electric potential map file (EMDB entry EMD-8116; Sirohi *et al.*, 2016 ▸) is 1.6 GB when stored using the CCP4 format. This amount of data is prohibitively large for display in a web browser. Therefore, a more efficient system for data delivery is even more crucial than for coordinates. The selective delivery of electron-density and electric potential maps was first introduced in the *LiteMol* suite. It includes *DensityServer*, which can adaptively downsample, slice and compress the volumetric data. For the Zika virus capsid data set, it can reduce the size to 1 MB while still maintaining visual fidelity (see Fig. 4 ▸).

In Mol*, *DensityServer* was superseded by *VolumeServer* (available to users at https://maps.rcsb.org/), which adds support for more data formats. Web tools that support *DensityServer*/*VolumeServer* are *JSmol* (Hanson *et al.*, 2013 ▸), the *LiteMol* suite and Mol*.

### 2.3. Data delivery: annotations   

Many tools and biological databases provide enriched annotations for macromolecular data. Some of them (for example, structure-validation data, as collected in wwPDB validation reports; Gore *et al.*, 2017 ▸) are part of the Protein Data Bank FTP site. Many others are collected together in PDBe-KB, which is a community-driven resource for structural and functional annotations. Specifically, PDBe-KB currently contains more than 500 million manually curated or predicted residue-level annotations for PDB structures obtained from close to 20 partner resources: for example, residue depths, binding-site predictions, ligand interactions, interaction interfaces, backbone flexibility predictions, solvent accessibility, kinase-target predictions, molecular channels, functional site predictions, druggable pocket predictions, energetic consequences of mutations, curated regulatory sites, curated post-translational modification (PTM) sites, curated catalytic sites, mutations in the human proteome, curated metal-binding sites and short linear motifs. PDBe-KB data are integrated with the core PDBe data in a graph database. Weekly snapshots of the graph database index are made available on the PDBe FTP area (ftp://ftp.ebi.ac.uk/pub/databases/msd/graphdb/). To ensure consistent and robust access to all of the PDBe-KB data, PDBe-KB also maintains a REST API (implemented in the Flask framework for Python), which includes 50 public endpoints. This API provides a source of annotation for web visualization tools.

### 2.4. Visualization: coordinate and experimental data   

*NGL* (Rose & Hildebrand, 2015 ▸), *LiteMol* and Mol* can provide interactive and highly responsive visualizations. Electron-density and electric potential maps can be displayed using *JSmol*, the *LiteMol* suite and Mol*. The *LiteMol* suite visualizes either experimental data for the whole structure (an overview, no details depicted) or for individual residues and their surroundings (only part of the data is shown). Mol* uses a similar approach to the *LiteMol* suite. *JSmol*, the *LiteMol* suite and Mol* all have support for *DensityServer*/*VolumeServer*. Mol* is the successor to the *NGL* and *LiteMol* viewers, combining the strengths of both viewers.

### 2.5. Visualization: annotations   

Annotation data include structural properties, physicochemical properties, charges and biological properties. They can be based on the whole structure or only a subset, such as the ligands.

**Figure 5:**
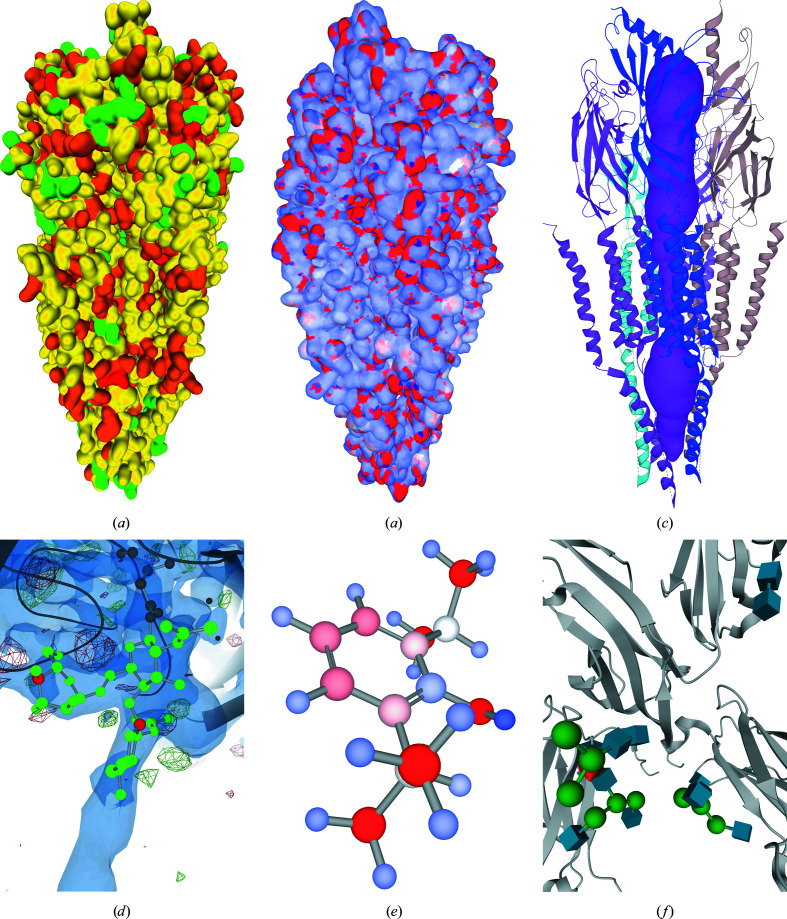
Visualization of whole structure annotation: (*a*) validation data from wwPDB validation reports (PDB entry 2bg9; Unwin, 2005 ▸), (*b*) partial atomic charges from *Atomic Charge Calculator II* (*ACC II*; Raček *et al.*, 2020 ▸) (PDB entry 2bg9) and (*c*) pore from ChannelsDB (Pravda *et al.*, 2018 ▸) (PDB entry 2bg9). Visualization of ligand annotation: (*d*) ligand-validation data from ValidatorDB (Sehnal, Svobodová Vařeková *et al.*, 2015 ▸) including electron densities (PDB entry 3d12; Xu *et al.*, 2008 ▸), (*e*) partial atomic charges on ligands from *ACC II* (PDB residue ID PFL) and (*f*) carbohydrate nomenclature visualization (Sehnal & Grant, 2019 ▸) (PDB entry 3sgj; Ferrara *et al.*, 2011 ▸).

Because validation annotation data are directly supported in the PDB archive, they are also integrated into the *LiteMol* suite, *i.e.* residues are coloured according to a value that describes the cumulative number of validation problems that occur in the residue (see Fig. 5 ▸ *a*). In this way, residue quality is also included in *JSmol* and *NGL*. Mol* offers even more detail: each residue can also be coloured according to several individual validation problems (atom clashes, Ramachandran outliers *etc.*). Moreover, the *LiteMol* suite and Mol* also integrate information about the quality of ligands (*e.g.* chirality problems; see Fig. 5 ▸ *d*). The *LiteMol* suite and Mol* also include nomenclature annotations, *i.e.* Carbohydrate Symbols (3D-SNFG; see Fig. 5 ▸ *f*).

External tools and data resources deliver other types of annotation data, and their visualization is performed on the individual web pages. Specifically, these resources integrate and customize a web visualization tool and utilize it for displaying niche data specific to that particular resource. This integration and customization can be very straightforward, such as merely colouring residues or atoms according to some property (for example partial atomic charge; see Figs. 5 ▸ *b* and 5 ▸ *f*), or may require some code extensions, such as showing defined protein surfaces, which is necessary for channel visualization (see Fig. 5 ▸ *c*). Other similar examples include *Fragalysis* (https://fragalysis.diamond.ac.uk), a web-based platform for fragment-based drug discovery, *ProteinPlus* (Fährrolfes *et al.*, 2017 ▸), which serves as a structure-based modelling support server, and 3*DBionotes-WS*, which automatically annotates biochemical and biomedical information onto structural models (Segura *et al.*, 2019 ▸).

### 2.6. Dynamic data and structure ensembles from molecular-dynamics simulations   

Since 2000, there have been multiple proof-of-concept studies (Meyer *et al.*, 2010 ▸) trying to enable the sharing of biomolecular simulation data. One of the surviving online databases of biomolecular trajectories from this era is MoDEL (Molecular Dynamics Extended Library; Meyer *et al.*, 2010 ▸; http://mmb.pcb.ub.es/MoDEL/), which pioneered the usage of a download capability for trajectories together with their metadata description with automatic analyses of the system (for example r.m.s.d. or contacts) and visualization using *Jmol* (Hanson, 2010 ▸) and non-interactive video. Decades later, all aspects of simulation data delivery have been enhanced by current transfer speeds, but mainly by new graphics possibilities owing to the development of WebGL (Khronos Group Inc, 2020 ▸).

First of all, online data-storage capacity increased to allow the storage of gigabytes of data daily. Molecular-dynamics trajectories are thus shared in general-purpose scientific data-storage systems such as Zenodo (https://zenodo.org/), OSF (Center for Open Science; https://osf.io) and FigShare (https://figshare.com), on the webpages of individual institutions or within specialized journals such as *Scientific Data* (Hoffmann *et al.*, 2020 ▸). This is the method that is used for the sharing of simulation data within the NMRlipids (Botan *et al.*, 2015 ▸) project and the current BioExcel/MolSSI COVID-19 repository (Amaro *et al.*, 2020 ▸; https://covid.molssi.org/simulations/). This approach allows data sharing, but it does not provide easy-to-use visualization, nor does it support any form of unified metadata describing the simulations.

Visualization can be provided based on the download and visualization of individual frames, such as with *MDsrv* (Tiemann *et al.*, 2017 ▸), *HTMol* (Carrillo-Tripp *et al.*, 2018 ▸), *JSmol* or even with generic 3D viewers such as *Autodesk*360 (https://a360.autodesk.com/). *Autodesk*360 was used for the visualization of *cellPACK* ensembles (Johnson *et al.*, 2016 ▸). *JSmol* is used in the BiGNAsim repository for the visualization of molecular-dynamics simulations of nucleic acids (Hospital *et al.*, 2016 ▸). However, *JSmol* is only capable of reading very few trajectory file formats and requires expert knowledge to use, and also has size limits. *MDsrv* together with *NGLviewer* (Rose & Hildebrand, 2015 ▸) was used in the novel GPCRmd platform (http://gpcrmd.org/), which offers insight into the 3D-GPCRome (analysis of the intrinsic flexibility of the structures of G-protein-coupled receptors; GPCRs), which are the primary targets for many drugs and endogenous signalling pathways (Rodríguez-Espigares *et al.*, 2020 ▸). To date, GPCRmd contains more than 580 molecular-dynamics trajectories along with an interactive on-site analysis provided by *MDtraj* (McGibbon *et al.*, 2015 ▸) and *Caver* 3.0 (Chovancova *et al.*, 2012 ▸), and custom scripts for more than 200 GPCR systems. GPCRmd thus allows comparative analyses of the whole GPCR protein family to be performed in an open, collaborative and reproducible manner. Furthermore, this is currently a goal of many approaches in the field of molecular simulation (Abraham *et al.*, 2019 ▸).

However, network capabilities limit making such approaches available on a general basis, as molecular-dynamics simulations can generate almost limitless amounts of data depending on the system size and sampling. Downloads of complete simulation data are thus slow owing to their size, which often can range from hundreds of megabytes to terabytes or more. Since the analysis of such data requires only a subset of all the generated data, it is probably the best option to transfer only the subset of data necessary for the analysis (*i.e.* visualization for insight and a list of critical molecular variables over time). A possible solution would be the utilization of selective data delivery that enables web visualization to speed up. *MDSrv* enables the selection and display of a specific frame and atomic selection. Mol* allows the animated visualization of the trajectory, and hence optimization of the data-delivery protocol via *ModelServer* allows the more efficient online visualization of molecular-dynamics trajectories.

## 3. Conclusion   

Data delivery and responsive web visualization of biomacromolecular structures, including their experimental data, annotations and data on dynamics, is a large challenge in the utilization of macromolecular structure data, especially since the sizes and numbers of snapshots of structures are still growing. To solve this complex challenge, relevant algorithms and software solutions have been developed and are in the process of further improvement. Specifically, selective data delivery, providing only the coordinate data necessary for visualization, was introduced in the *LiteMol* suite (using *CoordinateServer* and later improved by *ModelServer* in Mol*) and adopted by *NGL* and *JSmol*. In parallel, the selective delivery of volumetric data was implemented first in the *LiteMol* suite (via *DensityServer*) and then also in *NGL*. This was followed by the development of *VolumeServer*, which is integrated with Mol* and *JSmol*. Annotation data are collected into PDBe-KB and are accessible using its REST API. The visualization tools *LiteMol* suite, *NGL*, *JSmol* and Mol* benefit from these data-delivery services. These tools are integrated into many tools focused on providing structure annotations for specific domains. Structural ensembles can be visualized using *MDSrv* with *NGL* or Mol* to perform analysis of structure ensembles such as MD trajectories, and also support selective data delivery for its effective visualization, even for large systems. The future thus might lead to the establishment of online environments for structural data analysis based on cloud solutions, effectively speeding up their investigation.

## Footnotes

1 Source codes for the *Mol** *ModelServer* are available at https://github.com/molstar/molstar/tree/master/src/servers/model.
