# High-performance macromolecular data delivery and visualization for the web. Corrigendum

**DOI:** 10.1107/S205979832001606X

**Published:** 2021-01-01

**Authors:** David Sehnal, Radka Svobodová, Karel Berka, Alexander S. Rose, Stephen K. Burley, Sameer Velankar, Jaroslav Koča

**Affiliations:** a CEITEC – Central European Institute of Technology, Masaryk University, Kamenice 753/5, 625 00 Brno, Czech Republic; b National Centre for Biomolecular Research, Faculty of Science, Masaryk University, Kamenice 753/5, 625 00 Brno, Czech Republic; c Protein Data Bank in Europe (PDBe), European Molecular Biology Laboratory, European Bioinformatics Institute (EMBL–EBI), Wellcome Genome Campus, Hinxton CB10 1SD, United Kingdom; d Regional Centre of Advanced Technologies and Materials, Department of Physical Chemistry, Faculty of Science, Palacký University Olomouc, Šlechtitelů 241/27, 779 00 Olomouc, Czech Republic; e Research Collaboratory for Structural Bioinformatics (RCSB), San Diego Supercomputer Center, University of California San Diego, 9500 Gilman Drive, La Jolla, San Diego, CA 92093-0743, USA; f RCSB Protein Data Bank, Institute for Quantitative Biomedicine and Department of Chemistry and Chemical Biology, Rutgers, The State University of New Jersey, 174 Frelinghuysen Road, Piscataway, NJ 08854-8076, USA; g Cancer Institute of New Jersey, Rutgers, The State University of New Jersey, 195 Little Albany Street, New Brunswick, NJ 08903-2681, USA; h RCSB Protein Data Bank, San Diego Supercomputer Center and Skaggs School of Pharmacy and Pharmaceutical Sciences, University of California San Diego, 9500 Gilman Drive, La Jolla, CA 92093-0654, USA

**Keywords:** data delivery, visualization, macromolecules, corrigendum

## Abstract

Two citations in the article by Sehnal *et al.* [(2020), *Acta Cryst.* D**76**, 1167–1173] are corrected.

## Abstract 2

The article by Sehnal *et al.* [(2020), *Acta Cryst.* D**76**, 1167–1173] is corrected.

In the Introduction section of the article by Sehnal *et al.* (2020 ▸) two incorrrect citations were given for PDB-Dev. The correct citations are Vallat *et al.* (2018 ▸) and Burley *et al.* (2017 ▸).

## References

[bb1] Burley, S. K., Kurisu, G., Markley, J. L., Nakamura, H., Velankar, S., Berman, H. M., Sali, S., Schwede, T. & Trewhella, J. (2017). *Structure*, **25**, 1317–1318. 10.1016/j.str.2017.08.001 PMC5821105 28877501

[bb3] Sehnal, D., Svobodová, R., Berka, K., Rose, A. S., Burley, S. K., Velankar, S. & Koča, J. (2020). *Acta Cryst.* D**76**, 1167–1173. 10.1107/S2059798320014515 PMC7709201 33263322

[bb2] Vallat, B., Webb, B., Westbrook, J. D., Sali, A. & Berman, H. M. (2018). *Structure*, **26**, 894–904. 10.1016/j.str.2018.03.011 PMC5990459 29657133

